# Activation of the p53 signaling pathway by piRNA-MW557525 overexpression induces a G0/G1 phase arrest thus inhibiting neuroblastoma growth

**DOI:** 10.1186/s40001-023-01493-w

**Published:** 2023-11-08

**Authors:** Tao Mi, Xiaojun Tan, Zhang Wang, Zhaoxia Zhang, Liming Jin, Jinkui Wang, Mujie Li, Xin Wu, Dawei He

**Affiliations:** 1 Department of Urology; Chongqing Key Laboratory of Children Urogenital Development and Tissue Engineering; Ministry of Education Key Laboratory of Child Development and Disorders; National Clinical Research Center for Child Health and Disorders; China International Science and Technology Cooperation Base of Child Development and Critical Disorders; Chongqing Key Laboratory of Pediatrics, Children’s Hospital of Chongqing Medical University, Yuzhong District, Chongqing, 400014 People’s Republic of China; 2 Department of Urology, Nanchong Central Hospital, The Second Clinical Medical College, North Sichuan Medical College, Nanchong, 637000 Sichuan China

**Keywords:** piRNAs, piRNA-MW557525, Neuroblastoma, P53, Cell cycle

## Abstract

**Background:**

Neuroblastoma (NB) is the most common extracranial malignant solid tumor in children. Due to drug resistance to radiotherapy and chemotherapy, mainly due to the existence of cancer stem cells (CSCs), some children still have a poor prognosis. Therefore, researchers have focused their attention on CSCs. Our research group successfully constructed cancer stem cell-like cells named Piwil2-iCSCs by reprogramming human preputial fibroblasts (FBs) with the PIWIL2 gene in the early stage, and Piwil2-iCSCs were confirmed to induce the formation of embryonic tumors. PiRNAs, noncoding small RNAs that interact with PIWI proteins, play important roles in a variety of tumors. Therefore, our study aimed to explore the role of differentially expressed (DE) piRNAs derived from sequencing of Piwil2-iCSCs in NB.

**Methods:**

The DE piRNAs in Piwil2-iCSCs were screened using high-throughput sequencing and further verified in NB tissues and cells. An unknown piRNA, named piRNA-MW557525, showed obvious downregulation in NB. Thus we studied the effect of piRNA-MW557525 on the biological behavior of NB through in vitro and in vivo experiments. On this basis, we successfully constructed a stably transfected NB cell line overexpressing piRNA-MW557525 and performed transcriptome sequencing to further explore the mechanism of piRNA-MW557525 in NB.

**Results:**

In vitro, piRNA-MW557525 inhibited NB cell proliferation, migration and invasion and induced apoptosis; in vivo, piRNA-MW557525 significantly reduced the volume and weight of tumors and inhibited their proliferation, migration and invasion. piRNA-MW557525 overexpression induced G0/G1 phase arrest in NB cells via activation of the P53-P21-CDK2-Cyclin E signaling pathway thus inhibiting NB growth.

**Conclusions:**

Our findings show that piRNA-MW557525 functions as a tumor suppressor gene in NB and may serve as an innovative biomarker and possible therapeutic target for NB.

**Supplementary Information:**

The online version contains supplementary material available at 10.1186/s40001-023-01493-w.

## Introduction

Neuroblastoma (NB) is the most common extracranial malignant solid tumor in children, accounting for 7–8% of childhood malignancies, and > 90% of patients are diagnosed before the age of 10 [1]. In children aged 18 months or older with NB, the survival rate has improved to 40–50% with the current comprehensive treatment entailing surgery combined with chemoradiotherapy [2]. However, due to reasons such as resistance to radiotherapy and chemotherapy, some children still have a poor prognosis. Resistance to chemoradiotherapy is mainly due to the presence of cancer stem cells (CSCs), a small subset of cells in tumor tissue that are persistent and capable of initiating and spreading tumors [3, 4]. Unlike differentiated cancer cells, CSCs typically remain quiescent or have a relatively slow cell cycle, and after chemotherapy, they can re-engage in the cell cycle, whereas most differentiated cells experience death or cell cycle arrest [5]. Since the theory of CSCs was proposed, an increasing number of new reports on tumor recurrence, drug resistance and metastasis have been published. Therefore, researchers have focused their study on CSCs to identify effective therapeutic factors to target CSCs. However, the isolation and culture of CSCs is still very difficult, and thus, many researchers have focused on constructing CSCs through genetic reprogramming to solve this problem.

Aberrant overexpression of various embryonal genes and proto-oncogenes, including various cancer testis antigens (CTAs), is observed in malignant tumors. As a member of the CTA family, PIWIL2 is involved in initiation or maintenance of CSCs, thereby promoting tumorigenesis and malignant progression [6]. Our research group successfully constructed the ideal cancer-like stem cell model, Piwil2-iCSCs, by reprogramming human fibroblasts (FBs) with piwil2 in the early stage and confirmed that Piwil2-iCSCs can induce the formation of embryonic tumors in mice [7]. On the other hand, NB originates from neural crest cells mainly located in the adrenal glands, and these cells differentiate into adrenal chromaffin cells and sympathetic ganglion cells, which are also found in embryonal malignancies [8]. Therefore, we hypothesized that the DE genes, including mRNAs, piRNAs, and miRNAs, screened in Piwil2-iCSCs could also be used to study NB. PiRNAs are non-coding RNAs with a length of 26–31 nt bases. They exert their function by binding to Piwi proteins. It is interesting to note that while PIWIL genes encode piwil proteins rather than directly generating piRNA, the piRNA-PIWIL protein complex can generate additional piRNA through a ping-pong amplification mechanism [9]. PiRNA have been confirmed to play important roles in a variety of tumors. Downregulation of piR-001773 and piR-017184 significantly inhibited the growth of prostate cancer and may be therapeutic targets for prostate cancer. Serum piR-020619 and piR-020450 show strong potential as colorectal cancer-specific biomarkers for early detection [10, 11]. Thus, whether there is differential expression of piRNAs after PIWIL2 reprogramming and whether the DE piRNAs play important roles in the occurrence and development of NB deserve further study.

Our research group screened the DE piRNAs in Piwil2-iCSCs by high-throughput sequencing. We further verified the expression of these piRNAs in the tissues and cells of NB and found that the unreported piRNA-MW557525 was expressed at significantly low levels in NB tissues and cells. This sparked our interest in exploring the function of piRNA-MW557525. Therefore, we focused on piRNA-MW557525 as our research target and further analyzed the effect of piRNA-MW557525 on the biological behavior of NB and explored related mechanisms through in vitro and in vivo experiments to provide a new basis for the diagnosis and treatment of NB.

## Methods and materials

### Clinical samples

This study was approved by the Ethics Committee of Children's Hospital of Chongqing Medical University, and all patients and their parents signed informed consent forms before joining the study. A total of 41 NB tissues and 33 adjacent normal adrenal tissues were collected from patients undergoing urological surgery at the Children's Hospital of Chongqing Medical University. The detailed clinical data of the patients were summarized, including age, sex, site, INSS stage, prognosis, lymph node metastasis status and bone marrow metastasis status, survival state and Overall Survival (OS).

### Cell culture

FBs and Piwil2-iCSCs (provided by Chongqing Children's Urogenital Development and Tissue Engineering Key Laboratory) were cultured in Dulbecco's modified Eagle’s medium (DMEM) containing 10% fetal bovine serum. The cultures were then incubated at 37 °C in a 5% carbon dioxide atmosphere. The NB cell lines SK-N-SH, SH-SY5Y, SK-N-BE2, and IMR32 and the normal control cell line HEK-293 T were purchased from the Cell Bank of the Chinese Academy of Sciences (Shanghai, China). NB cell lines and HEK-293 T cells were cultured in DMEM supplemented with 10% fetal bovine serum and 1% penicillin streptomycin solution (Gibco, USA), and all cells were incubated at 37 °C with 5% CO_2_.

### Screening and validation of DE piRNAs

Total RNA was isolated from Piwi2-iCSCs and FBs, and piRNAs were screened by high-throughput sequencing. Two biological replicates were used for each group. Library construction and high-throughput sequencing were performed by LC Biotechnology Co., Ltd. (Hangzhou, China). Gene expression data of piRNAs were analyzed using R (R4.0.1) software and the edgeR software package. Genes with FPKM values lower than 3 in all samples were eliminated. *P*-value < 0.05 and |logFC|> 2 were used as screening thresholds.

The top 10 most upregulated and the top 10 most downregulated piRNAs in Piwi2-iCSCs were verified by RT-PCR (Additional file 1: Fig. S1), and the expression of piRNA-MW557525 was verified in NB tissue and NB cell lines. Total RNA was extracted from NB tissues and cell lines using TRIzol reagent. For piRNAs, the isolated RNA was reverse transcribed using a cDNA synthesis kit (TIANGEN, China). Then, the amount of cDNA was detected using SYBR Premix Ex Taq II, and the reaction was performed on a CFX96 Real-Time PCR detection system. The 20 DE piRNAs and U6 primers were synthesized by bioengineering organisms, and the specific sequences are shown in Table 1. All reactions were performed in triplicate. Finally, the 2−ΔΔCt method was used to calculate the relative expression of piRNAs, with U6 as the internal reference.

**Table 1:** The top 10 u-regulation and downregulation piRNA and U6 sequence-specific primers

Base sequence (5′–3′)	Primer sequences (5′–3′)
	Forward:
U6	CGCTTCACGAATTGCGTGTCAT
CCTTTCTGTGTGGAATTTGAATATCTGAAA	GCCTTTCTGTGTGGAATTTG
GGAGGTGATGAACTGTCTGAGCCTGACCT	GAGGTGATGAACTGTCTGAG
CCAGGGTTGATTCGGCTGATCTGGCTGGCT	GGGTTGATTCGGCTGATCT
TCTCGTGATGAAAACTCTGTCCAGTT	TCTCGTGATGAAAACTCTGTC
TAACTATGACTCTCTTAAGGTAGCCA	GCAGTAACTATGACTCTCTTAAGGT
GAGCCTCGGTTGGCCTCGGATAGCCGGTC	CGGTTGGCCTCGGATAG
ATGTTGGATCAGGACATCCCGATGGTGCAGCC	AGGACATCCCGATGGTG
CCAGGGTTGATTCGGCTGATCTGGCT	GGGTTGATTCGGCTGATCT
TAACTATGACTCTCTTAAGGTAGCC	GCAGTAACTATGACTCTCTTAAGGT
AACACCCTGATTGCTCCTGTCTGATT	ACACCCTGATTGCTCCT
TGAGTGTGTGTGTGTGAGTGTGTGA	GTGAGTGTGTGTGTGTGAG
ACAGTAGTCTGCACATTGGTTAAAA	CAGACAGTAGTCTGCACATTG
TACAGTAGTCTGCACATTGGTTAAA	CAGTACAGTAGTCTGCACATTG
GGTGAATGATGAACATGAACTTTCTGACC	GGTGAATGATGAACATGAACTTTC
GTGAATGATGAACATGAACTTTCTGACC	GGTGAATGATGAACATGAACTTTC
TGATTCCGGATCAGAAGATTGAGGGT	GTGATTCCGGATCAGAAGATTG
TGGTGTAATGGTTAGCACTCTGGACT	GTGGTGTAATGGTTAGCACTC
TGGTGTAATGGTTAGCACTCTGGACTCTGAAT	GTGGTGTAATGGTTAGCACTC
TGGACTCTGAATCCAGCGATCCGAGT	CAGTGGACTCTGAATCCAG
CAGCTACATCTGGCTACTGGGTCTC	CTACATCTGGCTACTGGGT

The Uni-piR qPCR primer was included in the kit

### piRNA-MW557525-transfected NB cells and construction of stably transfected strains overexpressing piRNA-MW557525

The piRNA-MW557525 inhibitor, piRNA-MW557525 mimic and corresponding negative controls(NC) were purchased from Shanghai GeneBio (Shanghai, China) and used to transfect SK-N-SH and SH-SY5Y cells (inhibitor group, inhibitor NC group, mimics group, mimic NC group). Cell transfection was performed using Lipofectamine 2000 (Invitrogen, Carlsbad, CA, USA) according to the manufacturer's protocol. The sequences of these oligonucleotides are listed in Table 2. Stable transfectants were constructed by transfecting SK-N-SH cells with lentivirus overexpressing piRNA-MW557525. The piRNA lentiviral particles and control vector (GFP lentivirus) were designed and purchased from Gene Corporation (Shanghai, China). First, cells were transfected with lentiviruses at different multiplicities of infection (MOI = 1, 10 and 100) and using different transfection reagents (mock, HiTransGA and SiTransGP) according to the manufacturers’ instructions. The transfected cells were collected 72 h after transfection, and the transfection efficiency was evaluated via RT–qPCR. Finally, GFP + (successfully transfected) cells were sorted using a fluorescence-activated cell sorting (FACS) flow cytometer (Becton Dickinson). The selected stable cells were cultured for subsequent experiments.

**Table 2:** The top 10 upregulation and downregulation piRNA and U6 sequence-specific primers

piRNA	Sequences (5′–3′)
Mimics	UGGAGGUGAUGAACUGUCUGAGCCUGACCUU
Mimics NC	UCACAACCUCCUAGAAAGAGUAGA
Inhibitor	AAGGUCAGGCUCAGACAGUUCAUCACCUCCA
Inhibitor NC	UCUACUCUUUCUAGGAGGUUGUGA

### Detection of cell proliferation ability

Cell proliferation ability was detected with cell counting kit-8 (CCK-8). A total of 3000 cells/well were plated in 96-well plates and then grouped for transfection. After 0, 24, 48 and 72 h of transfection, the medium was discarded, and 10 μL of CCK-8 solution and 90 µl of fresh medium were added to each well and incubated with cells at 37 °C for an additional 2 h. The absorbance value (optical density, OD value) at 450 nm was measured with a microplate reader (Bio–Rad, USA).

### Detection of apoptosis ability

The apoptosis ability of cells was detected using an Annexin V-FITC/PI apoptosis detection kit (CWBIO, Beijing, China). Cells were collected and washed twice with cold PBS. Then, 500 μL of binding buffer was added to resuspend the cells at a concentration of 1 × 10^6^/ml. Then, 5 μL of Annexin V/FITC and 5 μL PI were added to each group of cells for staining, and the cells were incubated in the dark at room temperature for 15 min. The stained cells were immediately detected via flow cytometry (BD Bioscience, USA), and the apoptosis rate was analyzed using FlowJo software.

### Detection of cell migration and invasion ability

A wound-healing assay was used to assess the migratory capacity of cells. Cells (2 × 10^5^ cells) were seeded in 6-well plates to achieve 90% confluency. The monolayer was then scratched with a 10 µL sterile pipette tip to simulate a wound. After removal of detached cells with PBS, the adherent cells were cultured in serum-free medium for 48 h. After 0, 24, and 48 h of culture, cell migration was recorded under a microscope (Nikon, Tokyo, Japan), and the width of the scratch was measured using ImageJ software.

For invasion experiments, 60 µl of Matrigel (1:5 dilution in serum-free DMEM, BD Biosciences, USA) was prelaid on the bottom of a transwell chamber with an 8-µm pore polycarbonate membrane. Cells (5 × 10^4^ per well) resuspended in 200 µl serum-free medium after transfection were plated in the upper chamber, and 800 µl medium containing 10% fetal bovine serum was added to the lower chamber. After incubation for 24 h, the cells in the upper chamber were gently wiped away with a cotton swab, and the invading cells were fixed with 4% paraformaldehyde, stained with 0.5% crystal violet, and then randomly selected and counted in 5 fields under a microscope (K10587; Nikon).

### Transcriptome sequencing and bioinformatics analysis of the piRNA-MW557525-overexpressing stable transgenic strain and control group cells

To further study the mechanism by which piRNA-MW557525 inhibits the growth of NB, transcriptome sequencing of the stably transfected strain and control group cells was performed. Three samples were used per group. After removal of genes with low-expression, differential analysis was performed using the R language edgeR package. *P* values less than 0.05 and logFD greater than 1 were considered statistically significant. Kyoto Encyclopedia of Genes and Genomes (KEGG) pathway analysis, Gene Ontology (GO) functional enrichment analysis and Gene Set Enrichment Analysis were performed on differentially expressed mRNAs in R software using the clusterProfiler software package. Pathways with a corrected *P* value less than 0.05 were considered significant.

### Cell cycle

To investigate the influence of piRNA-MW557525 on the cell cycle, cells from the mimic group, mimic NC group, inhibit group, and inhibit NC group were employed for cell cycle analysis using a cell cycle assay kit (Dojindo, Japan). Simultaneously, the mimics group, mimics NC group, and mimics + p53 inhibitor (pifithrin-α hydrobromide (MCE), 40 µg/10 ml) group were utilized to delineate the impact of the P53 signaling pathway on the cell cycle. The experimental process is briefly described as follows: Cells (1 sample: 1–2 × 10^6^ cells) were collected, washed twice with cold PBS, and fixed with 70% ethanol overnight. The next day, the cells were washed with PBS and then stained with Working Solution (1 sample: 500 μl Assay Buffer + 25 μl PI Solution + 2.5 μl RNase Solution) for 30 min at 4 °C and 30 min at 37 °C in the dark. Cell cycle distribution was assessed using flow cytometry (BD Biosciences, USA) and analyzed using ModFitLT software.

### Western blotting

After transfection, the total protein from the cells was treated with the p53 inhibitor MCE (40 µmol), and tumor tissues were lysed in RIPA buffer containing 1% phenylmethylsulfonyl fluoride and detected with a BCA detection kit (BCA, Thermo, Shanghai, China) to determine the protein concentration. The same amount of protein (20 µg) was separated by 10% sodium dodecyl sulfate–polyacrylamide gel electrophoresis (SDS–PAGE) and transferred to a polyvinylidene fluoride (PVDF) membrane (Micropore, USA). The membranes were then blocked in 5% nonfat milk for 1 h and incubated with primary antibody overnight at 4 °C. The primary antibodies targeted the following proteins: MMP2 (1:1000, Proteintech, China), MMP9 (1:1000, Proteintech, China), PCNA (1:1000, ZENBIO, China), Bcl-2 (1:1000, ZENBIO, China), Bax (1:3000, Proteintech, China), cleaved caspase-3 (1:1000, Proteintech, China), p21 (1:500, Wanleibio, China), cyclin E (1:500, Wanleibio, China), CDK2 (1:300, Wanleibio, China), p53 (1:1000, Proteintech, China), and GAPDH (1:5000, ZENBIO, China). Finally, the membrane was incubated with the corresponding secondary antibodies at room temperature for 2 h, and positive bands were detected by chemiluminescence.

### Animal models

All procedures in this study were approved by the Institutional Animal Care and Use Committee of Chongqing Medical University, and nude mice (4 weeks old, male) were purchased from the Animal Research Center of Chongqing Medical University (Chongqing, China). We randomly divided the mice into 3 groups: the piRNA-MW557525 stable overexpression group (mimics group), lentivirus negative control group (mimics -NC group) and blank control group. To form tumor xenografts, 2 × 10^6^ SK-N-SH cells were injected subcutaneously into the armpits of nude mice (6 mice/group). The long and short diameters of the tumors were measured with a vernier caliper once every 5 days, and the tumor volume was calculated (volume calculation formula: long diameter × short diameter 2 × 0.5) until the 15th day. After 15 days, 75 mg/kg d-luciferin (Xenogen) was subcutaneously injected while the mice were under isoflurane inhalation anesthesia, and bioluminescence images were obtained using an IVIS imaging system (Xenogen). The analysis was performed using in vivo imaging software (Xenogen), which assessed photon flux in a region of interest (ROI) drawn around a region of bioluminescence intensity. Finally, the mice were sacrificed, and the tumors were collected and weighed. A portion of the tumor specimens was fixed with 4% paraformaldehyde, and the remaining portion was frozen in liquid nitrogen for later use.

### Immunofluorescence

NB tumor tissue specimens were fixed with 4% paraformaldehyde, embedded in paraffin, and cut into 4 μm thick sections. Sections were deparaffinized in water, heated in 100 °C citrate buffer (pH 6.0) for 15 min to recover antigens, and then blocked with 5% bovine serum albumin (BSA) for 1 h. The cells were incubated with primary antibody overnight at 4 °C, washed 3 times with PBS, incubated with fluorescent secondary antibody for 1 h in the dark, washed 3 times with PBS and subjected to Hoechst staining for 30 min to observe cell nuclei. Finally, the slices were mounted under a fluorescence microscope and photographed (Olympus Crop, Tokyo, Japan).The cells were placed in a 24-well plate prelaid with cell slides. After grouping, they were washed 3 times with PBS, fixed with 4% paraformaldehyde for half an hour, blocked with 0.2% Triton X-100 and 0.5% BSA for 1 h, and incubated with primary antibodies overnight, consistent with tissue immunofluorescence practices.The primary antibodies used for immunofluorescence (targeting PCNA, MMP2, MMP9, Bax, BCL2, cleaved caspase-3) were the same as those used for western blotting (WB) but diluted with 0.5% BSA at a ratio of 1:200.

### Statistics

Data are presented as the mean ± standard deviation (SD), and statistical analysis was performed using GraphPad Prism software (GraphPad 6.0) and R software (R.4.0.1). Student's t test was used to determine whether differences between two groups were statistically significant. One-way ANOVA was used to compare the data of more than three groups, and *P* < 0.05 was considered to be statistically significant. Difference analysis of genes was undergoing by edgeR package. KM curve was used to evaluate the relationship between piRNA expression and the OS. A log-rank test was used to compare the survival differences between different groups. All experiments were repeated at least 3 times independently.

## Results

### The expression of piRNA-MW557525 was downregulated in NB and correlated with prognosis

**Fig. 1:**
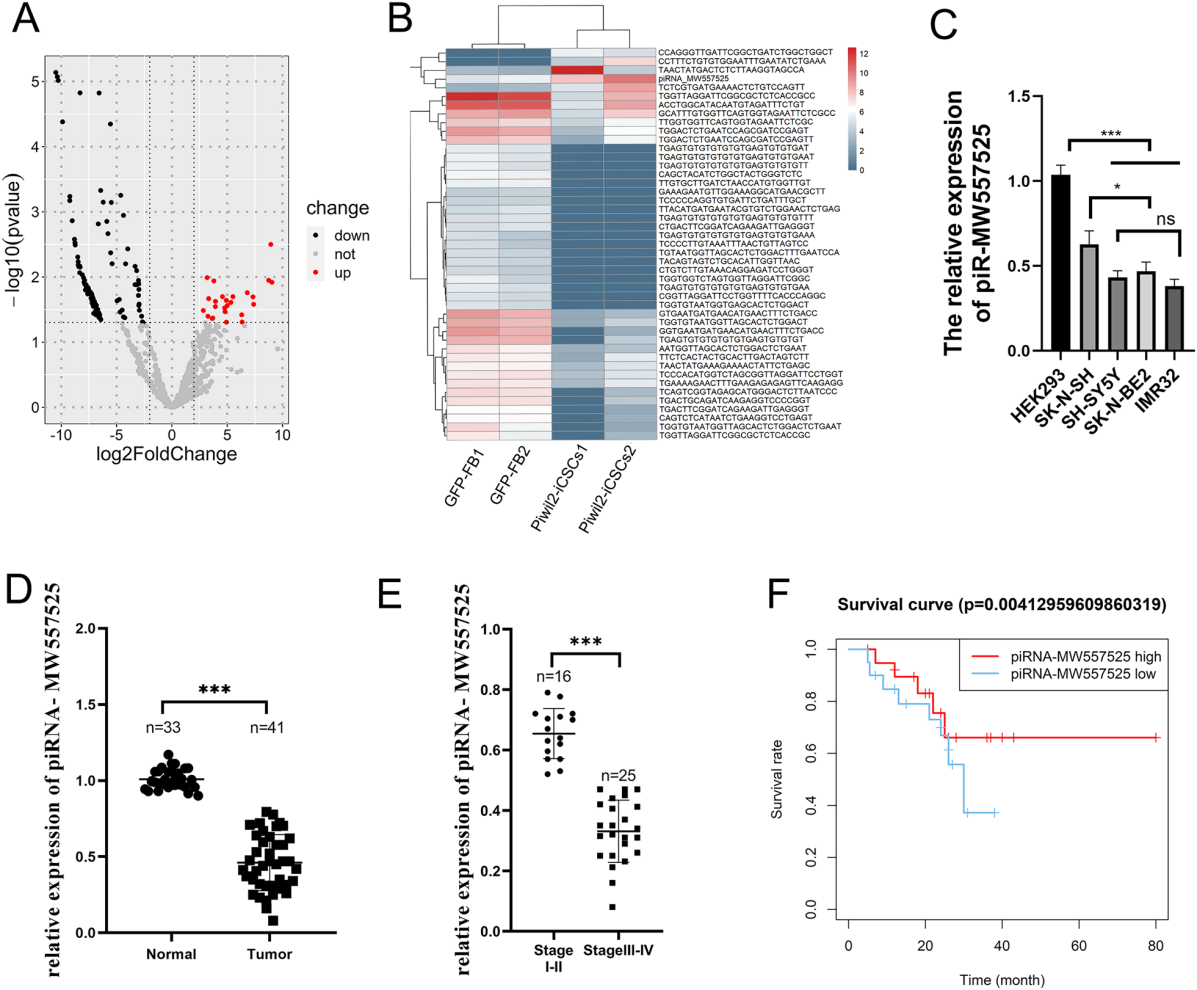
Differentially expressed piRNAs and the correlation between piRNA-MW557525 and clinical features. **A** Volcano map of the differential piRNAs in PIWIL2-iCSC compared with GFP-FB by high-throughput sequencing. **B** Heat map of the 50 most differentially expressed piRNAs. **C** piRNA-MW557525 expression level in NB cell lines (SK-N-SH, SH-SY5Y, SK-N-BE2, IMR32) compared with HEK293 according to RT-qPCR. **D** Relative piRNA-MW557525 expression was determined in 41 NB tissues and 33 adjacent nontumor tissues by RT-qPCR. **E** Expression of piRNA-MW557525 in different stages of neuroblastoma according to RT-qPCR. **F** The relationship between piRNA-MW557525 and patient survival. ns *P* > 005; *P < 0.05; ***P < 0.001

We first detected the expression profiles of piRNAs in PIWIL2-iCSCs using RNA sequencing and identified DE piRNAs in PIWIL2-iCSCs compared with GFP-FB cells (Fig. 1A, B). The verification results for the 10 obvious up-regulated expressed piRNAs showed that piRNA-MW557525 was the the most significantly one. (Additional file 1: Fig. S1). The expression of piRNA-MW557525 was further verified in NB cells and tissues. However, the results showed that the expression of piRNA-MW557525 was significantly reduced in NB cells compared with the level in the normal control cell line HEK-293 (Fig. 1C). Compared with adrenal medulla tissue, piRNA-MW557525 was significantly downregulated in 41 NB tissues (Fig. 1D). To further explore the clinical significance of piRNA-MW557525 in NB, we analyzed the relationship between the piRNA-MW557525 level and the age, sex, tumor location, ISNN stage, prognosis, lymph node metastasis status and bone marrow metastasis status of NB patients. As shown in Table 3, the expression of piRNA-MW557525 was significantly correlated with the TNM stage, and OS(*P* < 0.05) (Fig. 1E, F). However, there was no significant correlation between piRNA-MW557525 and other clinical parameters.

**Table 3:** The relationship between piRNA-MW557525 and the clinicopathological characteristics of NB patients

Characteristics	*n*	Percentage (%)	piRNA-MW557525 relative expression *P*
Clinicopathological features			0.0***
Adjacent noncancerous tissues	33	80.5	
NB	41	100	
Age			0.524
≥ 1.5	13	31.7	
< 1.5	28	68.3	
Sex			0.912
Male	18	43.9	
Female	23	56.1	
Location			0.692
Left retroperitoneal	22	53.7	
Right retroperitoneal	18	43.9	
Others	1	2.4	
INSS			< 0.001
I + II	16	39.0	
III + IV	25	61.0	
Prognostic type			< 0.01
FH	13	31.7	
UFH	28	68.3	
Lymphatic metastasis			0.653
Yes	27	65.9	
No	14	34.1	
Bone marrow metastasis			0.725
Yes	31	75.6	
No	10	24.4	

NB, neuroblastoma; INSS, International Neuroblastoma Staging System; FH, favorable histology; UFH, unfavorable histology

### piRNA-MW557525 inhibits the proliferation and promotes apoptosis of SK-N-SH and SH-SY5Y cells

**Fig. 2:**
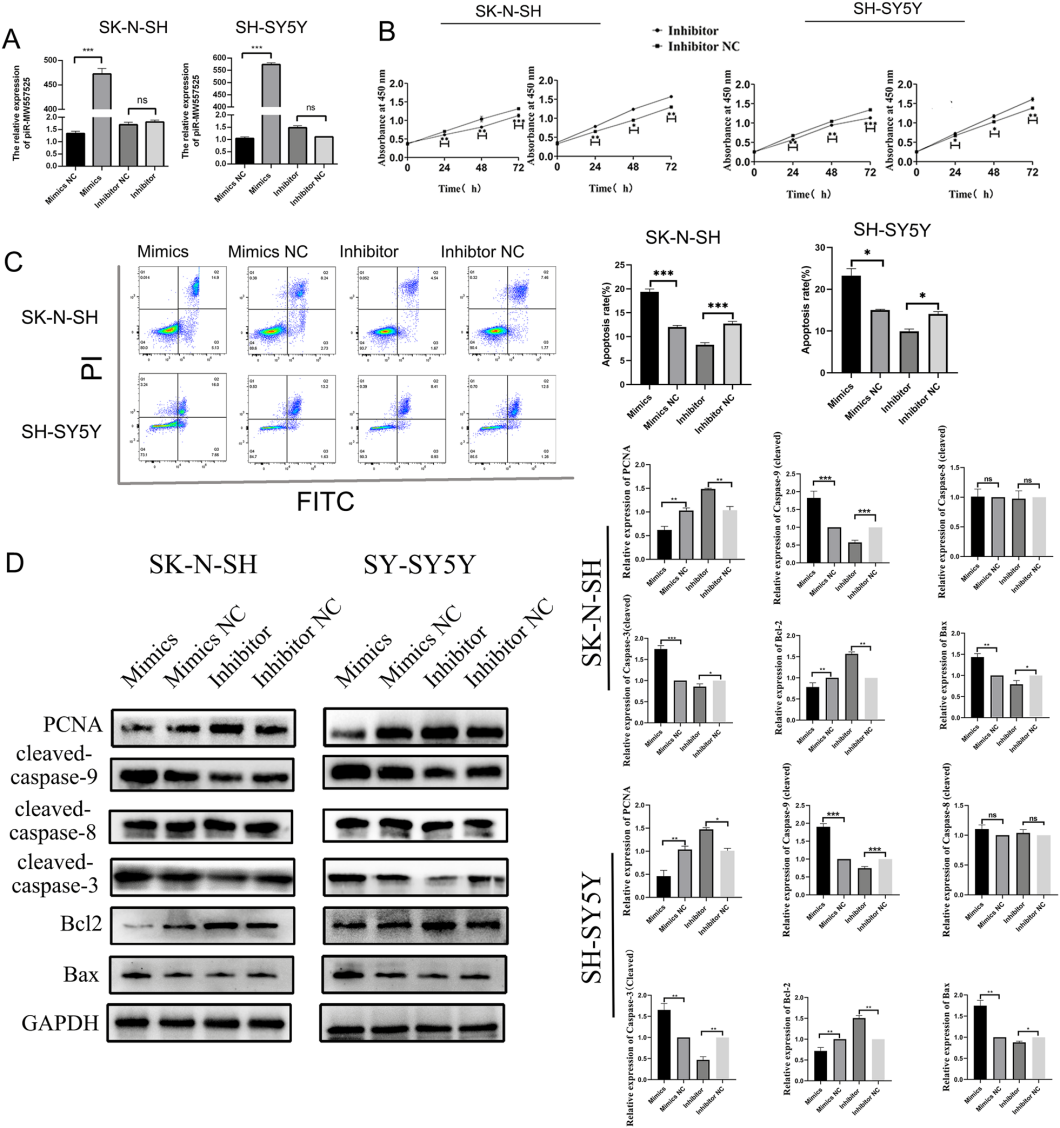
piRNA-MW557525 inhibits the proliferation and promoted the apoptosis of NB cells. **A** piRNA-MW557525 expression was measured to confirm the mimics and inhibitor efficiency in SK-N-SH and SH-SY5Y cells. **B** CCK-8 assay was performed to determine the proliferation of NB cells transfected with piRNA-MW557525 mimics, inhibitor and NC. **C** Flow cytometry analysis was performed to determine the apoptosis after piRNA-MW557525 mimic, inhibitor or NC transfection of SK-N-SH and SH-SY5Y cells. **D** The relative expression of PCNA, Bcl-2, Bax and cleaved caspase-3/8/9 was detected by western blot analysis. ns *P* > 0.05, *P < 0.05, **P < 0.01, ***P < 0.001. vs. piRNA-NC

We investigated the effect of piRNA-MW557525 on the biological behavior of NB. First, we successfully overexpressed piRNA-MW557525 using liposome technology (Fig. 2A). The results of CCK8 assays showed that overexpression of piRNA-MW557525 significantly inhibited the proliferation of SK-N-SH and SH-SY5Y cells, while downregulation of piRNA-MW557525 significantly promoted cell proliferation (Fig. 2B). Next, we investigated the role of piRNA-MW557525 in SK-N-SH and SH-SY5Y cell apoptosis. Flow cytometry results revealed that in SK-N-SH and SH-SY5Y cells, overexpression of piRNA-MW557525 significantly increased the number of apoptotic cells compared with negative control cells, while downregulation of piRNA-MW557525 inhibited apoptosis in cells (Fig. 2C). We further verified the expression levels of proliferation and apoptosis-related proteins. WB results showed that overexpression of piRNA-MW557525 promoted the expression of cleaved caspase-3/9 and Bax proteins and inhibited the expression of the PCNA and Bcl-2; downregulation of piRNA-MW557525 led to the opposite results (Fig. 2D). However, the effect on exogenous apoptosis protein cleaved caspase-8 was not obvious. The above results demonstrated that piRNA-MW557525 inhibits the proliferation and promoted endogenous apoptosis in SK-N-SH and SH-SY5Y cells.

### piRNA-MW557525 inhibits the migration and invasion of SK-N-SH and SH-SY5Y cells

**Fig. 3:**
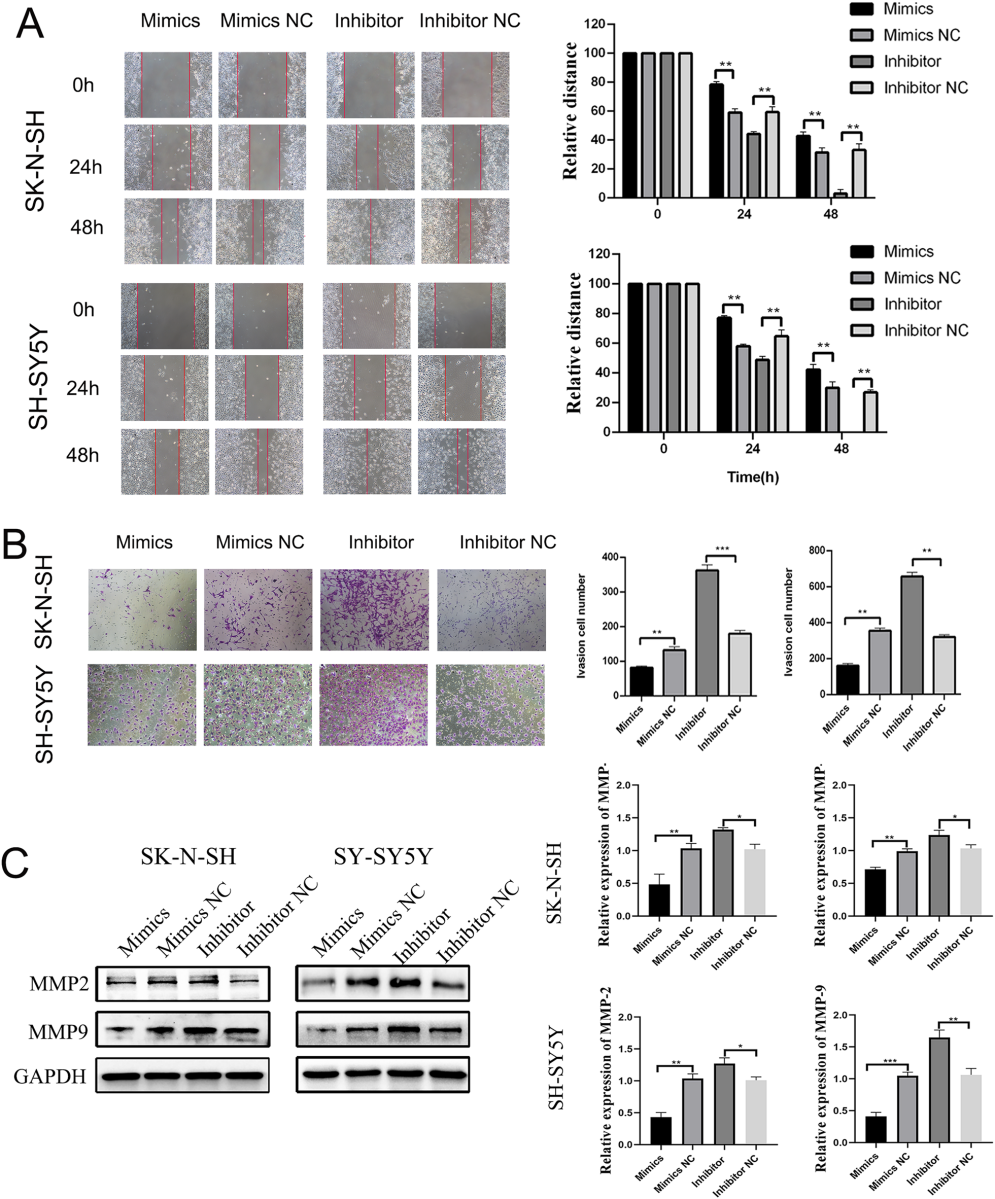
PiRNA-MW557525 suppressed NB cell migration, and invasion. **A** Wound healing tests was performed to assess the migration of SK-N-SH and SH-SY5Y cells transfected with piRNA-MW557525 mimic, inhibitor and NC. **B** Transwell analysis was used to determine the invasion of SK-N-SH and SH-SY5Y cells transfected with piRNA-MW557525 mimic, inhibitor and NC. **C** The protein levels of MMP2 and MMP9 were detected after piRNA-MW557525 mimics, inhibitor and corresponding NC transfection. *P < 0.05, **P < 0.01, ***P < 0.001. vs. piRNA-NC

Scratch experiments showed that overexpression of piRNA-MW557525 significantly inhibited the migration of SK-N-SH and SH-SY5Y cells, while downregulation of piRNA-MW557525 promoted cell migration (Fig. 3A). Transwell assay results showed that overexpression of piRNA-MW557525 significantly inhibited the invasion of SK-N-SH and SH-SY5Y cells, while downregulation of piRNA-MW557525 promoted cell invasion (Fig. 3B). In addition, WB results displayed that overexpression of piRNA-MW557525 reduced the expression levels migration- and invasion-related proteins MMP2 and MMP9 in NB cells, while downregulation of piRNA-MW55752525 promoted the expression of these proteins (Fig. 3C). The above results proved that piRNA-MW557525 inhibited the migration and invasion of SK-N-SH and SH-SY5Y cells.

### piRNA-MW557525 inhibits tumor growth in vivo

**Fig. 4:**
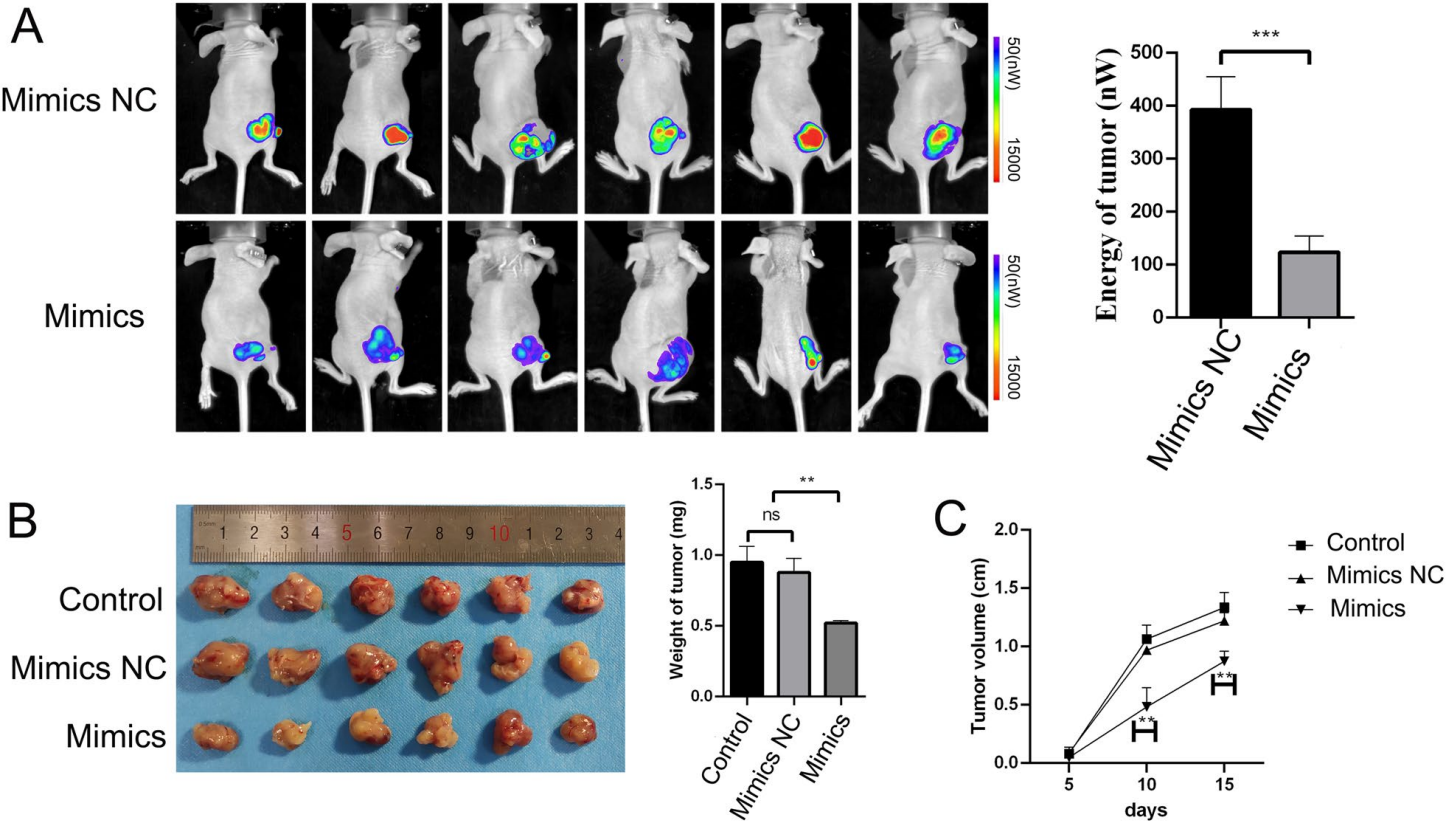
piRNA-MW557525 inhibited tumor growth in vivo. **A** Representative bioluminescent images were acquired after injection with piRNA-MW557525 mimics and mimics NC SK-N-SH cells (n = 5 for each group). **B** Quantitative analysis of the weight of tumors and xenografts resected from each group of BALB/c nude mice are shown. **C** The tumor volumes of mice were measured at 5, 10 and 15 days after injection, and growth curves were drawn. Ns *P* > 0.05, **P < 0.01, ***P < 0.001

To investigate the impact of piRNA-MW557525 on in vivo tumor growth, we employed NB cells(mimics group, mimics NC group, blank control group) for subcutaneous tumor xenografts. On the 15th day, we conducted in vivo imaging of the mice, which revealed reduced fluorescence intensity in the piRNA-MW557525 overexpression group (Fig. 4A). Subsequently, upon tumor extraction, we observed that the piRNA-MW557525 overexpression group exhibited smaller tumor volumes and weights (Fig. 4B). Ex vivo measurements of subcutaneous tumors in mice indicated significant differences as early as the 10th day (Fig. 4C). These findings provide compelling evidence that piRNA-MW557525 significantly suppresses in vivo tumor growth.

**Fig. 5:**
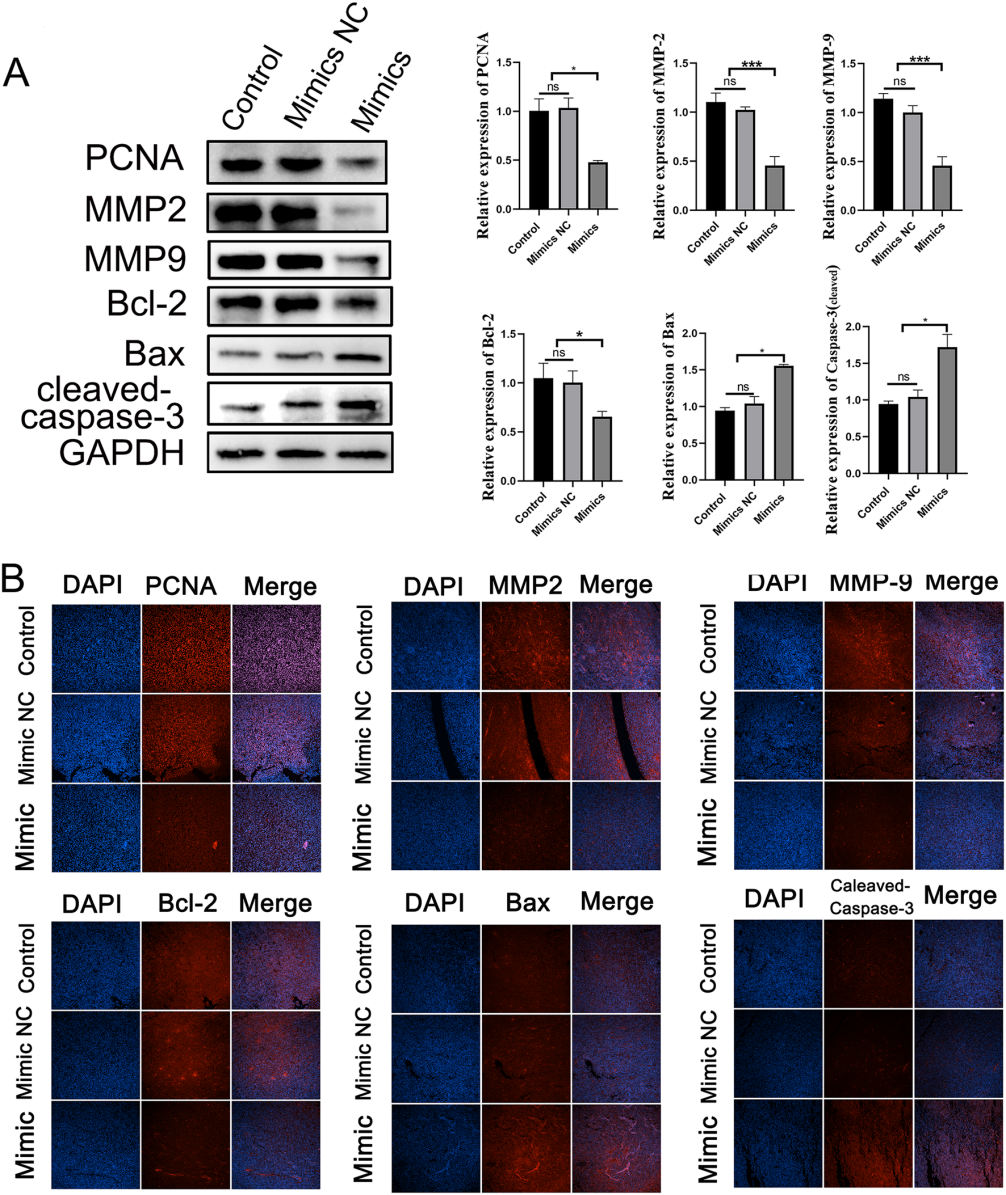
piRNA-MW557525 reduced the proliferation-, apoptosis- and invasion-related indicators in NB xenografts. **A** The protein levels of PCNA, MMP2, MMP9, Bcl-2, Bax, cleaved caspase-3 in tumors of nude mice were detected by WB, piRNA-MW557525 mimics and NC cells. **B** Immunofluorescence staining of the proliferation-, apoptosis- and invasion-related indicators. ns *P* > 0.05; *P < 0.05; ***P < 0.001. vs. piRNA-NC

We further assessed the expression of markers related to proliferation, apoptosis, and invasion in tumor tissues using immunofluorescence and WB analyses. The WB results indicated that the overexpression of piRNA-MW557525 significantly downregulated the expression of proliferation- and invasion-promoting proteins, such as PCNA, MMP2, and MMP9, when compared to the control and mimic NC groups. Simultaneously, the overexpression of piRNA-MW557525 promoted apoptosis of tumor cells in vivo, as evidenced by the up-regulation of apoptosis-related proteins, cleaved caspase-3 and Bax, and the downregulation of the apoptosis-inhibiting protein, BCL-2 (see Fig. 5A). Importantly, the immunofluorescence results exhibited consistent expression trends with the WB results. (Fig. 5B).

### piRNA-MW557525 induces cell cycle arrest in G0/G1 phase by activating the p53 signaling pathway

**Fig. 6:**
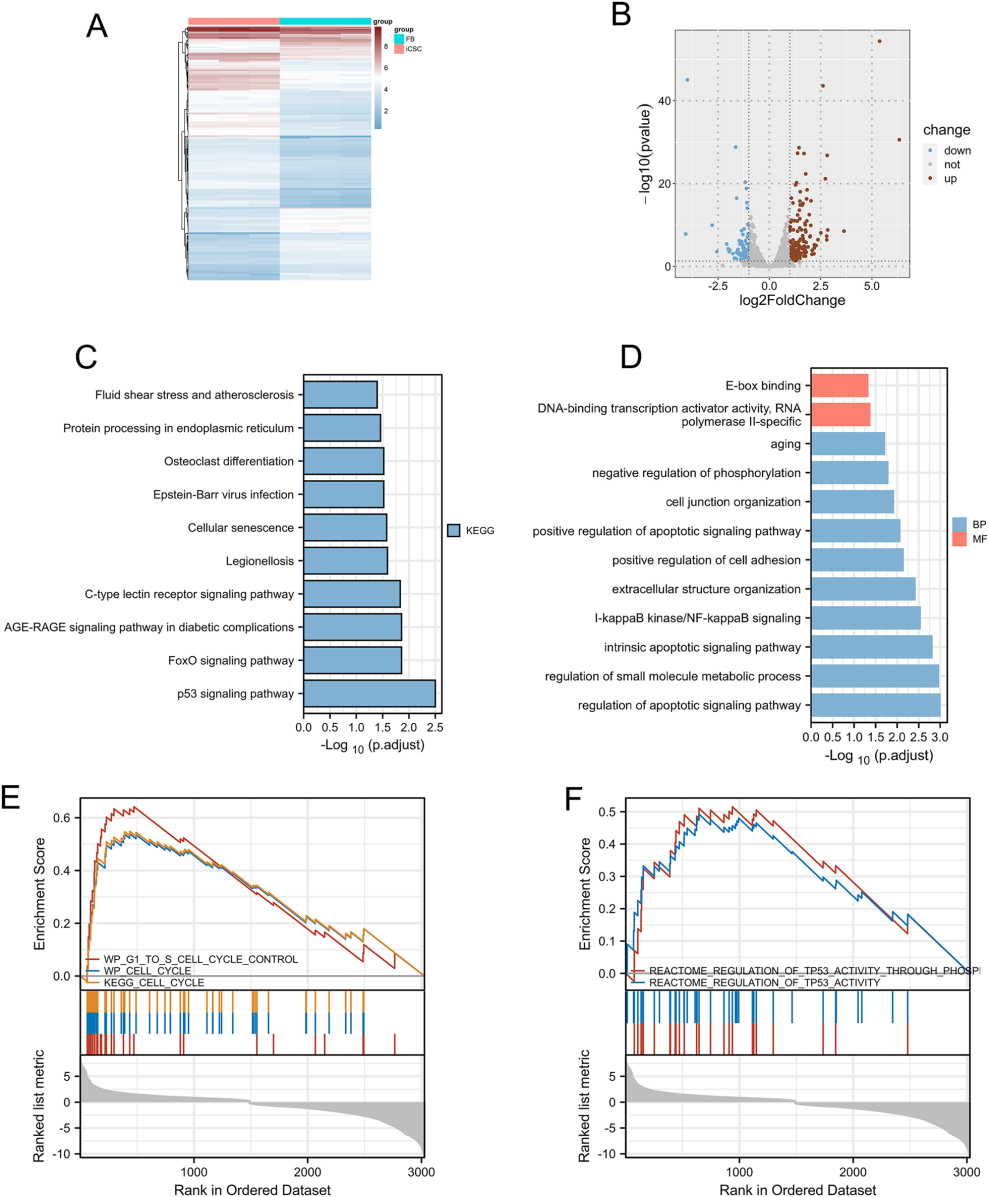
Difference analysis of the mRNA between mimics NC and mimics groups and enrichment analysis. **A**, **B** Heat maps and volcanic maps of different expressed genes. **C**, **D** KEGG and GO enrichment analysis of the different expressed genes. **E**, **F** Gene Set Enrichment Analysis (GSEA)

The constructed stable transgenic piRNA-MW557525-overexpressing strain and the control group cells were subjected to transcriptome sequencing, and a total of 254 significantly differentially expressed genes were screened, of which 89 genes were downregulated and 165 genes were up-regulated (Fig. 6A, B). KEGG analysis results showed that the p53 signaling pathway, foxo signaling pathway were enriched (Fig. 6C). GO analysis results showed apoptosis were enriched (Fig. 6D). GSEA results showed that the P53 pathway and cell circle pathway was activated in the piRNA-MW557525 overexpression group (Fig. 6E, F). All in all, the bioinformatics analysis results suggest that overexpression of piRNA-MW557525 can lead to activation of the p53 signaling pathway, which is a classic cell cycle-related pathway.

**Fig. 7:**
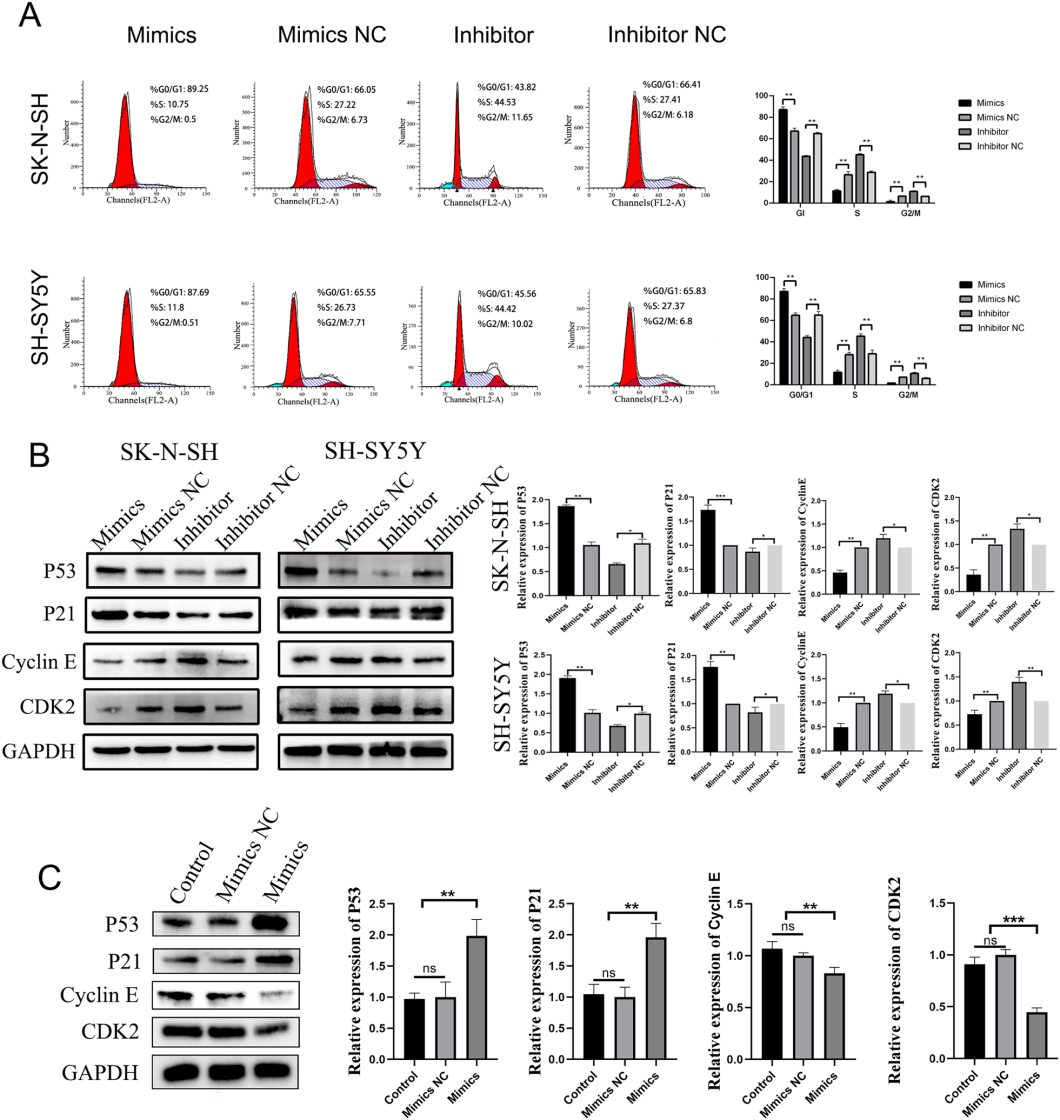
Effect of piRNA-MW557525 on the cell cycle of NB cells and the expression of cycle-related proteins. **A** The cell phases of SK-N-SH and SH-SY5Y cells were analyzed by flow cytometry after transfection with piRNA-MW557525 mimics, inhibitor and corresponding NC. **B** The protein levels of p53, p21, cyclin E and CDK2 were detected using western blotting after piRNA-MW557525 transfection in vitro. **C** Cell cycle-related protein in tumors of nude mice were detected by WB. ns *P* > 0.05; *P < 0.05; **P < 0.01; ***P < 0.001. vs. piRNA-NC

**Fig. 8:**
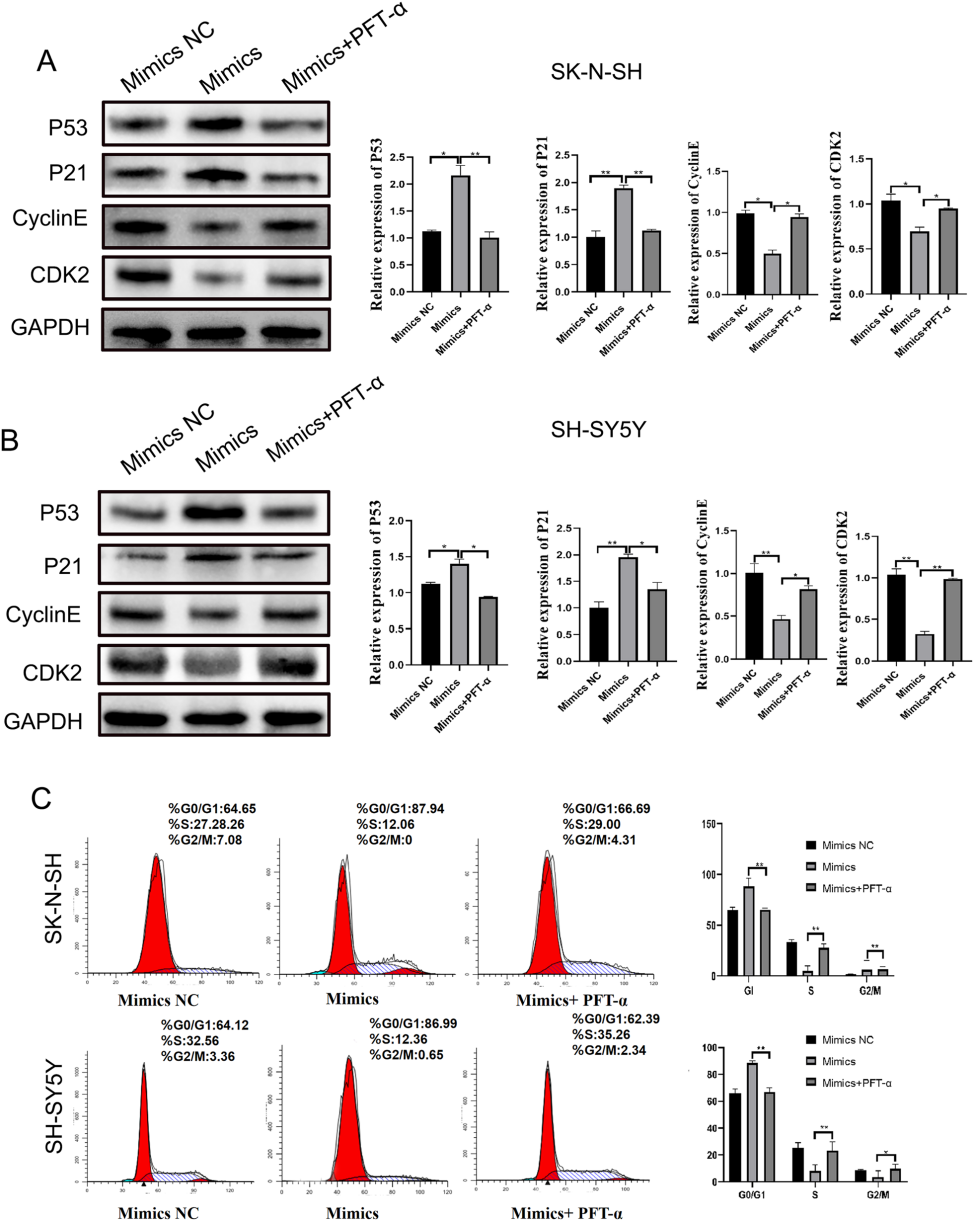
P53 inhibitors rescue cell cycle arrest from piRNA-MW557525. **A**, **B** Expression of P53 pathway and cell cycle-related proteins after the addition of P53 inhibitors. **C** The cell phases of SK-N-SH and SH-SY5Y cells were analyzed by flow cytometry in the miminc NC, mimincs, and mimics + PFT-α group

To further verify whether piRNA-MW557525 causes cell cycle arrest through the p53 pathway, we examined the cell cycle via flow cytometry. The results showed that overexpression of piRNA-MW557525 resulted in a significant increase in the proportion of cells in G0/G1 phase compared with the control group, while downregulation of piRNA-MW557525 resulted in a significant decrease in the proportion of cells in G0/G1 phase. In addition, overexpression of piRNA-MW557525 resulted in a significant decrease in the proportion of cells in S and G2/M phases, while downregulation of piRNA-MW557525 resulted in a significant increase in the proportion of cells in S and G2/M phases compared with the control group (Fig. 7A). In addition, we examined the p53 signaling pathway and the expression of cell cycle-related proteins, including p53, p21, cyclin E, and CDK2 in NB cells and tumors of nude mice via WB. The results showed that piRNA-MW557525 promoted the expression of p53 and p21 and inhibited the expression levels of cyclin E and CDK2 (Fig. 7B, C). The protein expression level of the p53 signaling pathway was detected after further addition of a p53 inhibitor. The results showed that the p53 inhibitor decreased the expression of p53 and p21 and increased the expression levels of cyclin E and CDK2 compared with levels in the mimic group (Fig. 8A, B). After addition of the p53 inhibitor, the cell cycle was detected again. The results proved that the ratio of cells in G0/G1 phase in the p53 inhibitor group was lower than that in the mimic group (Fig. 8C). The above results identified that piRNA-MW557525 induces cell cycle arrest in G0/G1 phase by activating the p53 signaling pathway.

## Discussion

Neuroblastoma is a heterogeneous solid tumor of the sympathetic nervous system, most commonly occurs in the abdomen, and accounts for approximately 15% of childhood cancer-related mortality [12]. Patients with low-risk NB typically receive minimal treatment, and some children are cured by surgery alone or may experience spontaneous tumor regression [13, 14]. Patients with low-risk NB have a good prognosis, with a 5-year survival rate of more than 90%; however, 60% of patients have high-risk NB, and the prognosis for such patients remains poor [15], with a 5-year survival rate of less than 50% [16, 17]. The reasons for tumor refractoriness may be related to metastasis, recurrence and drug resistance, and the previous view was that tumor metastasis, recurrence and drug resistance were due to the existence of CSCs. CSCs are capable of self-renewal and are found in most liquid and solid cancer types. Current chemotherapeutic drugs can kill most tumor cells, but CSCs are not sensitive to chemotherapeutic drugs and are considered to be the key cause of tumor onset, expansion, drug resistance, recurrence and metastasis [18]. Therefore, an increasing number of researchers use CSCs as research objects to find new potential targets that can be used for tumor diagnosis and treatment. Based on this, our research group constructed a cancer-like stem cell model, Piwil2-iCSCs, by reprogramming FBs with the PIWIL2 gene and confirmed that these cells can induce embryonic-derived tumors. NB is also an embryonic tumor. We expect to use the Piwil2-iCSCs as a cell model to screen diagnostic and therapeutic markers for embryonic-derived tumors. PIWIL2, also known as cancer/testis antigen, has been found to be highly expressed in a variety of cancer types, such as prostate, colorectal, breast, cervical, gastric, and ovarian cancer [19–22]. PIWIL2 is also a potent inhibitor of apoptosis, acts as a molecular marker of precancerous stem cells and promotes tumorigenesis [23]. piRNAs are noncoding small RNAs that interact with PIWI proteins. Abnormal expression of piRNAs is associated with a variety of cancer types and may play pro-cancer or anticancer roles in cancer occurrence, development and metastasis [24, 25]. Therefore, we further screened out DE piRNAs in Piwil2-iCSCs to identify new potential targets for the treatment of NB.

Our results revealed that the expression of piRNA-MW557525 was significantly lower in NB tissues and that the expression level in the third and fourth stages was lower than that in the first and second stages. In fact, it's an interesting phenomenon that this piRNA is up-regulated in Piwil2-iCSCs, but downregulated in neuroblastoma. This indicates that piRNA may play different roles in different stages of tumor, just like the TGF-β, which acts as a tumor suppressor in the early stage of cancer but is an oncogene in the late stage [26]. However, the specific mechanism needs to be further explored. Anyway, Survival analysis showed that piRNA-MW557525 was a favorable prognostic factor. Moreover, the expression of piRNA-MW557525 was confirmed to be negatively correlated with NB malignancy. Compared with normal control cells, piRNA-MW557525 was also expressed at low levels in NB cells. Through a series of in vitro and in vivo experiments, we confirmed that piRNA-MW557525 can inhibit the proliferation, migration and invasion of NB cells and can induce apoptosis to inhibit tumor growth. Through transcriptome sequencing, we further found that the p53 signaling pathway and cell cycle arrest were regulated by piRNA-MW557525.

As a pivotal tumor suppressor, the transcription factor p53 exerts a fundamental role in tumor suppression, with its primary mechanisms historically attributed to the induction of apoptosis and the imposition of cell cycle arrest [27, 28]. The inhibition of tumorigenesis via p53-mediated G0/G1 cell cycle arrest has been well-established across a diverse spectrum of malignancies [29–31]. In our study, we conducted a comprehensive assessment of cell cycle-related parameters utilizing flow cytometry and Western blot analysis. Our findings demonstrated that the overexpression of piRNA-MW557525 triggers the activation of the p53 pathway, leading to the modulation of key regulators, including P21, CDK2, and cyclin E, subsequently culminating in G0/G1 cell cycle arrest. Notably, the imposition of P53 inhibitors effectively rescinds the piRNA-MW557525-induced cell cycle arrest, underscoring the regulatory role of piRNA-MW557525 in neuroblastoma cell cycle progression through P53 activation.

The findings may be useful for the treatment of neuroblastoma. In particular, targeting the P53 signaling pathway emerges as an appealing strategy for the treatment of neuroblastoma. For example, the murine double minute 2 (MDM2) oncogene is frequently overexpressed in neuroblastoma, and it can bind to the P53 protein, thereby suppressing the anti-cancer effects of the P53 signaling pathway [32, 33]. The application of MDM2 inhibitors to disrupt the P53-MDM2 interaction has displayed promising efficacy, especially in patients exhibiting a paucity of P53 mutations. Interestingly, the MDM2 gene can be positively regulated by the P53 signaling pathway [32, 33]. Considering the capacity of the piRNA-MW557525 to activate the P53 pathway, the exploration of potential synergistic effects arising from the co-administration of piRNA-MW557525 with MDM2 inhibitors presents a promising and novel avenue for further investigation.

## Conclusion

Our research has uncovered a previously unreported piRNA, named piRNA-MW557525, which serves as a promising prognostic marker in neuroblastoma. We have substantiated that piRNA-MW557525 can induce G0/G1 phase arrest through the p53 signaling pathway, thereby inhibiting the progression of neuroblastoma.

### Supplementary Information

**Additional file 1: Figure S1.** RT-PCR verified the 10 most obvious up-regulation and downregulation piRNA in PIWIL2-iCSC and GFP-FB. **A** The downregulated piRNAs. **B** The upregulated piRNAs.

## Data Availability

The datasets used or analyzed during the current study are available from the corresponding author on reasonable request.
