# Supplementary figures and images for: Activation of the p53 signaling pathway by piRNA-MW557525 overexpression induces a G0/G1 phase arrest thus inhibiting neuroblastoma growth
Source: Eur J Med Res. 2023 Nov 8;28:503. doi: 10.1186/s40001-023-01493-w (PMC10631185; doi:10.1186/s40001-023-01493-w)

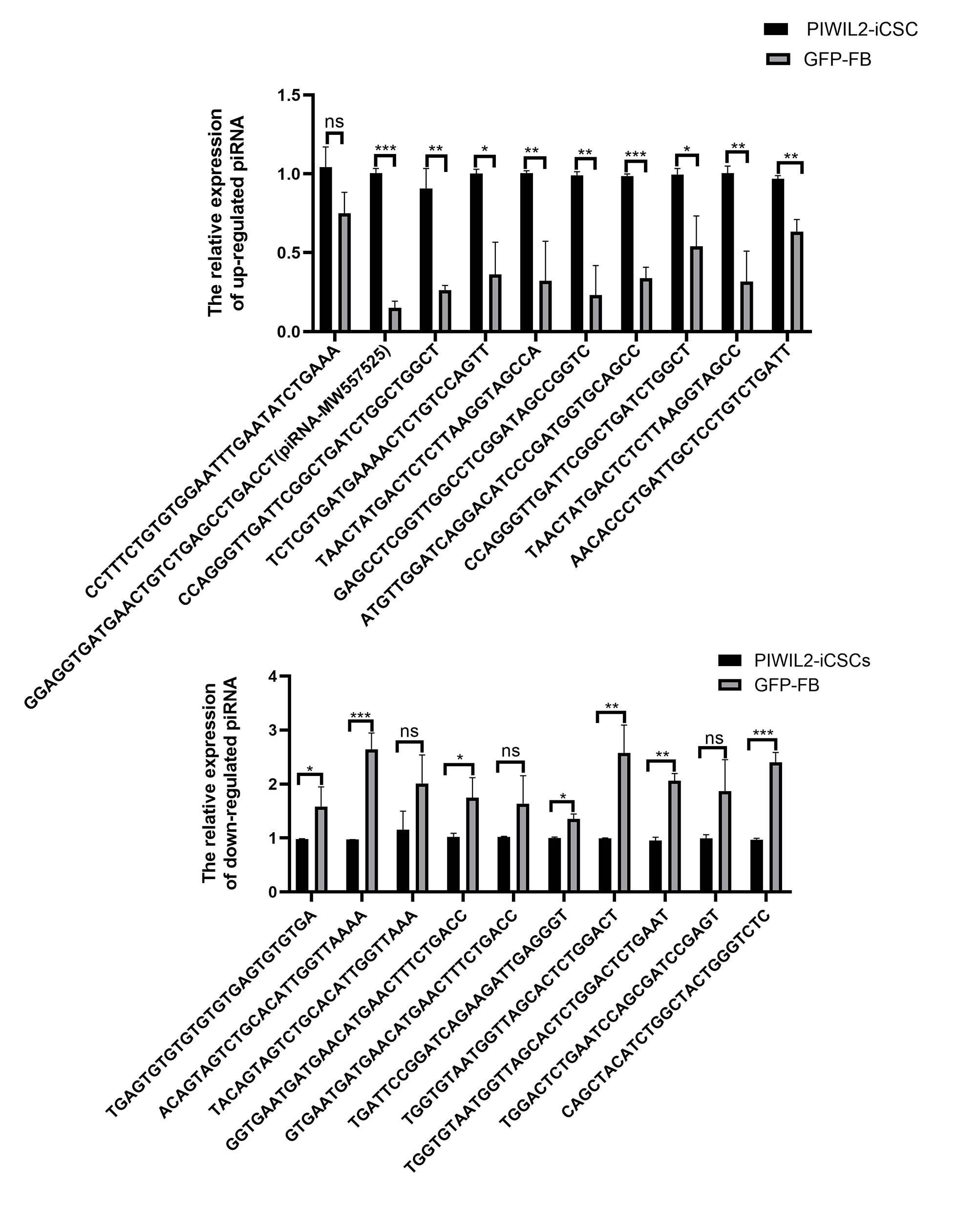

Supplement: Supplementary file 1 — Additional file 1: Figure S1. RT-PCR verified the 10 most obvious up-regulation and downregulation piRNA in PIWIL2-iCSC and GFP-FB. A The downregulated piRNAs. B The upregulated piRNAs. [file 40001_2023_1493_MOESM1_ESM.tif]
